# Fly Ash Modified Coalmine Solid Wastes for Stabilization of Trace Metals in Mining Damaged Land Reclamation: A Case Study in Xuzhou Coalmine Area

**DOI:** 10.3390/ijerph15102317

**Published:** 2018-10-21

**Authors:** Jiu Huang, Peng Wang, Chaorong Xu, Zhuangzhuang Zhu

**Affiliations:** 1 School of Environment Science and Spatial Informatics, China University of Mining and Technology, 221116 Xuzhou, China; jhuang@cumt.edu.cn (J.H.); crxu@cumt.edu.cn (C.X.); 2 State Key Laboratory for Geomechnics and Deep Underground Engineering, 221116 Xuzhou, China; pwang@cumt.edu.cn

**Keywords:** mining gangues, subsidence backfill, occurrence states transformation, element migration

## Abstract

In China, coalmine wastes, such as gangues, are used for reclamation of mining subsided land. However, as waste rocks, gangues contain several trace metal elements, which could be released under natural weathering and hydrodynamic leaching effects and then migrate into the reclamed soil layer. However, it is very difficult to find adequate other backfill materials for substitution of gangues. In this paper, we present a novel method and case study to restrict the migration ability of trace metal elements in gangues by using another kind of coalmine solid waste—fly ashes from coal combustion. In this study, fly ashes were mixed with gangues in different mass proportions 1:0.2, 1:0.4, 1:0.6 and 1:0.8 as new designed backfill materials. Due to the help of fly ash, the occurrence states of studied trace metal elements were greatly changed, and their releasing and migration ability under hydrodynamic leaching effect were also significantly restricted. In this research seven trace metal elements in gangues Cu, Zn, Pb, Cd, Cr, Mn and Ni were studied by using soil column hydrodynamical leaching method and simulated precipitation for one year. The results show that under the driving of natural precipitation trace metal elements were generally transported deep inside the reconstructed land base, i.e., far away from soil layer and most of the trace metal elements were transformed into a bonded state, or combined in inert occurrence states, especially the residual state. With this method, the migration activities of tested trace metal elements were greatly restricted and the environmental potential risk could be significantly reduced.

## 1. Introduction

China is the biggest coal production and consumption country in the world. Coal occupies more than 70% domestic primary energy consumption in China, which covers about 75% primary fuels for industries and more than 90% primary fuels for civil use. Furthermore, in China, it is believed that coal will continued to be used as the main energy source for a long period in future [1,2,3,4]. Most coal is produced through underground mining in China, which makes use of a huge land area. Until now, there are more than 4.3 × 10^5^ hm^2^ mining subsidence area in China currently. Under actual technologies, with a coal yield of 10^4^ t there are about 0.2 hm^2^ subsidence of land resource. The annual increment of mining subsidence area is about 5 × 10^4^ hm^2^ [2,3,4]. Meanwhile, coal mining has also generated huge amount of solid wastes, mainly waste rocks (coal gangues). Conventionally the coalmine gangues are disposed in dumps. At present, in China there are about 4.5 billion tons of coalmine solid wastes are stored in dumps: worse still is that the dumps keep increasing around 0.95 billion tons/year. At present, there are totally about 1500 coalmine waste dumps in China, which occupy more than 8 × 10^4^ hm^2^ land areas [5,6,7,8,9]. Mining subsidence and dumps destroy land resources and make them to be unavailable for any uses. Therefore, since the 1980s in China mining solid wastes had been used for reclamation of mining subsidence area, which is called backfill reclamation, which has been proved to be effective both in remediation of subsided land and disposal of mining solid wastes. Mining solid wastes consists mostly of waste rocks, which are produced during coal production and coal processing. Gangues take 15–40% mass of raw coals after wash-refining [10,11,12]. Coalmine wastes have huge yields in local areas e.g., in Xuzhou City, which has a coal mining history of more than 130 years; only huge amounts of gangues could fulfill the demands of backfill materials in large subsided area [1,2,13,14,15]. The subsided land reclamation could be divided into three processes: “reshaping”, “resoiling” and “replanting” [1,2,4,5,6,7]. For backfill reclamation, the mining solid wastes are only used for “reshaping” of subsided land, i.e., they constitute the base material of overlaying soil layers to upraise the surface to achieve ground level, on which the plants are rehabilitated. Gangues are rock materials, they are suitable for reconstruction of land base aggregate. However, for base reconstruction, fine materials, such as sands, are also necessary to ensure air permeability and water retention ability [7,10,12,14]. However, quantity of sands is limited according to regions, they are also relative too expensive. Together with coalmine there are always coal combustion power plants which could supply fly ashes with adequate amount as backfill material, fly ashes have very fine particle sizes and large surface areas.

Gangues and fly ash also contain several kinds of trace metals such like lead (Pb), cadmium (Cd), chromium (Cr), manganese (Mn), nickel (Ni), copper (Cu), zinc (Zn) etc., which are always hazardous to overlaying soil layers. In China, the “Directives of land resource reclamation” had regulated that among use types of reclaimed land, the agricultural use has the highest priority [2,4,5,6,7,13,14]. Related researchers have pointed out that there are risks of the trace metal elements releasing from mining wastes, as well as probabilities of migration into the plants and crops under certain circumstance conditions of reconstructed soil layers [4,5,12,13,16,17,18]. Trace metal elements could bring great threaten to human health. Pd and Cr, for example, Pd could lead to anemia and cause damage to the nervous system; Cr could destroy renal function, cause osteomalacia or even a cancer [19,20,21,22,23]. Therefore, most of the related researches concentrate on the release and migration behaviors of trace metals in reclaimed land. Their results have pointed out that trace metal elements in soil are much more difficult to migrate than in air and water, since the soil organic and inorganic colloidal particles have intensive adsorption and chelation effects on trace metals, which strongly limit their migration [2,5,8,16]. Most of the research in trace metal elements’ migration has pointed out that the release and migration behaviors are actuated by hydrodynamic leaching behavior, i.e., the soaking effect of groundwater and the leaching effect of precipitation [2,4,14,16,17,18,19,20,21]. According to previous studies, some of them show that there were low risks on trace metal elements’ migration [2,5,11]; the most common results demonstrate that with specific methods the potentials of trace metal contaminations could be effectively restricted and controlled [2,5,12,16,17,18,19,20,21,22,23,24,25]; and some of them reported that there were always high contaminations or potential of threatens [26,27,28]. O’Conner et al. [15,18] has reported that with method of adsorption by using biochar material could achieve perfect solidification of trace metals, but in our situation, it is impossible to obtain adequate biochar for several tens of square kilometer area. Wang et al. [20,21] have verified that with binding materials like MgO, several kinds of trace metals could be successfully stabilized. Weber et al. [22] and Żołnierz et al. [23] also reported that soil could be reconstructed by using fly ash over long periods. Actually, fly ash contains high proportion of metal oxides like MgO, which could be a nice material for trace metal binding. According to the studies of [21,22] on tremendous yield of fly ash from coal combustion power plants, the quantity requirements for large area reclamation could be guaranteed. However, fly ash comes from coal combustion, it contains also trace metals from coals. What will happen if the coal gangues are used together with fly ash in land reclamation? It is still a question to be studied. Most arguments in previous studies concentrated on the occurrence states variation of trace metal elements under natural hydrodynamic processes [29,30,31,32]. Variation of trace metals’ contents could not represent their migration potential, we should also consider the overall occurrence states changing, i.e., the migration abilities of hazardous trace metals. To our knowledge, no work has been reported on trace metals’ occurrence states’ transformation in reclaimed land and soil, especially regarding reclamation by using fly ash modified mining waste rock materials.

The occurrence state of trace elements usually refers to their binding and combination states, i.e., the presence of trace elements [33]. As people pay more and more attention to the environment, the research on the states of trace elements has received much more attention. The potential harmfulness, toxicity, migration and transformation of hazardous trace elements into environment has become a key points in research field. In addition to total amount of elements, occurrence state is also a key issue. There is no authoritative and unified classification of trace elements’ occurrence states. For coalmine solid wastes, the occurrence states of hazardous trace elements are very complicated [32,33,34,35]. The ways in which they combined with other substances and minerals determine their releasing and migration abilities into environment. Furthermore, their occurrence states could also be converted with the variation of physical-chemical conditions in soil and geological mediums.

Several kinds of methods have been developed for study of occurrence state, such as chemical sequential extraction, diffusive gradients in thin-films (DGT), spectral analysis, low temperature ignition, etc. Methods like DGT, which was commonly used in detection of trace metals in water and sediments, it is not suitable for analysis of trace metals in solid materials like coal gangue [36,37]; spectral analysis like nuclear magnetic resonance (NMR), electronic spectroscopy, were direct methods for trace metal detection, which were suitable for determining trace metal contents in coal with high precision; however, for occurrence states it was not accurate enough [38]; low temperature ignition was also a common method, which has similar limitation to spectral analysis in detection of occurrence states [39]. Therefore, for rock materials coming from coalmines in this research, we need to study series of substances and minerals combined with trace metals in different occurrence states, the method of sequential extraction is the most appropriate one. In recent years, the sequential extraction method has been widely used in the study of trace elements’ occurrence states. This method is able to examine occurrence states in high accuracy. It dispels solid samples into specific solutions under certain chemical conditions, and then determines how trace elements’ concentrations in the solution could represent their occurrence state in solid materials. With the modern measurement technologies, the sequential extraction method has achieved very high accuracy, especially together with mass spectroscopy method. Herein we present a method with occurrence states study by using sequential extraction to analyze the environmental risks on releasing and migration of trace metal elements from gangue-fly ash combined backfill materials. This study was designed according to an actual coalmine subsidence area near Xuzhou city, which will be soon reclaimed by using coalmine solid wastes.

## 2. Materials and Methods

### 2.1. Study Area and Targets

**Figure 1:**
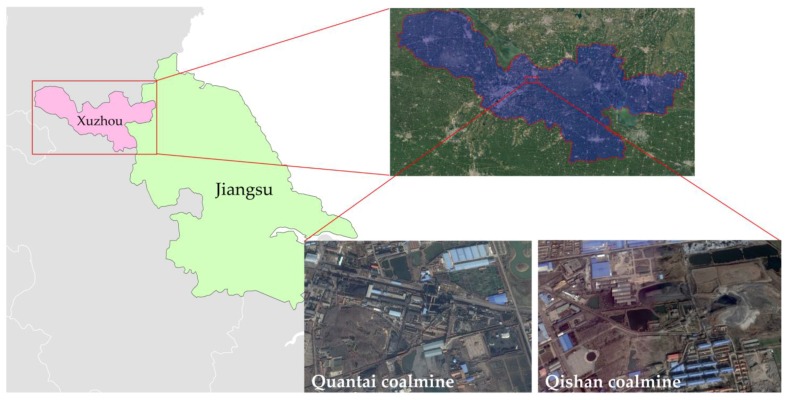
Location of Qishan-Quantai coalmines in Xuzhou City, Jiangsu Province.

This research is based on a coalmine subsidence area on the east of the Xuzhou City, which locates around the Qishan–Quantai coalmine region of Xuzhou Coalmine Group (34°20′01″ N, 117°24′20″ E), which is shown in Figure 1 as follows. At the end of 2017, all coalmines in this area were closed. The total subsidence area is about 8.2 km^2^, with average depth about 4.2 m. Local government has planned to restore the subsided area for agricultural land. The reclamation will be implemented with backfill method by mainly using of coalmine solid wastes. After reclamation the groundwater level was designed to be −2.0 m [2,14], and the thickness of the reclaimed soil layer was designed to be 20–30 cm [7]. Therefore, most of the backfilled coalmine wastes will be submerged in groundwater, and the distance between groundwater level and reconstructed soil layer is planned to be about 1.7–1.8 m [33,35]. As we have mentioned, the migration ability of trace metal elements in water is much stronger than in soil, and with the draining of precipitation, the migration of trace metals happens inside the backfill material layer from the bottom of soil layer to the groundwater. In addition, the key point to this study is the occurrence state transformation and migration of trace metal elements under natural precipitation.

### 2.2. Standard Methods

The studied backfill coalmine waste materials included gangue and fly ash were collected from coalmine and coal combustion power plants of Quantai–Qishan. Pure gangue has excellent performance as aggregate material but it has too high porosity and low water retention. Pure fly ash has too high pH value which always leads to soil slab, which strongly limits the water permeation. Hence in actual the gangue and fly ash are designed to be used with proportioning. Commonly there are several mass proportions between gangue and fly ash used, they are 1:0.2, 1:0.4, 1:0.6 and 1:0.8 according to different requirements in reclamation, respectively. Therefore, in this study we also study the occurrence state transformation and migration of trace metal elements in backfill materials with these above mentioned four mass proportions. According to the study purpose, five occurrence states of hazardous trace metals were studied to describe their migration abilities and their potential risks to the local environment:

(1) Exchangeable state

The exchangeable state is understood in most cases as the ionic state of adsorption-desorption effects of water. This state is the most active state to be transformed in the environment and result of great impact.

(2) Carbonates binding state

Elements that are capable to precipitate or co-precipitate as carbonate crystal, which can be released by mild acids, they are referred to be carbonates binding state. The pH value has significant effect on the migration ability of elements in this state, and acidic water can easily leach it. When the pH value decreased, the migration ability and biological activity of trace elements in this state will increase.

(3) Iron—manganese oxides combined state

Iron/manganese oxides or iron/manganese colloids can absorb or encapsulate trace elements in the sediment, such a state is called iron-manganese oxides binding states. Normally these kinds of binding states are stable, but the trace elements could be released under reductive conditions. This state is a reducible state.

(4) Organic sulfide binding state

Elements with some states can only be extracted and released by using strong oxidants such as hydrogen peroxide and sodium pyrophosphate (Na_4_P_2_O_7_); during the extraction process, these oxidants oxidize organics and sulfides in coal gangue samples. These elements can be co-precipitated with sulfide minerals or can be complexed with humic acids, fatty acids, alkanes, and other organic substances in the precipitate. Elements in this state are called organic sulfide binding states. The ions of harmful elements can produce concretion with the oxides, and the oxides themselves can also be transformed into particle capsules or inter-particle cements, which are due to their extremely strong adsorption. In the laboratory, the vast majority used oxidation is 30% H_2_O_2_ solution, and then the extraction with ammonium acetate solution.

(5) Residual state

In addition to above mentioned four states, in backfill material there are certain trace metal binding substances with particularly high stability; therefore, they have no opportunity to be released and transported into environment. This kind of binding state can only be extracted with extreme conditions, like per-chloric acid or mixed strong acids as extractants. This binding state of trace metals is called residual state. In the laboratory, strong acid or mixed strong acid is usually used for the degradation and analysis of residual state elements.

Sequential extraction is an appropriate method to study the occurrence states’ of trace elements.

### 2.3. Digestion Analysis of Trace Metal Elements Content in Backfill Materials

As an important basic of our study, the total trace elements’ content in designed backfill material must be quantitative determined. Here we used chemical digestion analysis. The specific operations of experiment were as follows:

(1) Preparations:

We prepared 10% dilute nitric acid solution into a bucket and then submerged vessels to be used in this experiment in dilute nitric acid for 48 h, then we cleaned the vessels with deionized water. Then they were dried in a drying oven.

(2) Experiment pretreatment:

Gangue material was crushed with a hammer mill, and 400 g crushed coal gangue powder were sampled and then fine grinded to particle size less than 0.1 mm. Then every 100 g of each coal gangue powder were fully mixed with sampled fly ashes, according to the mass proportions between coal gangue and fly ash of 1:0.2, 1:0.4, 1:0.6, 1:0.8, respectively. Then we took 2 g of each sample for testing.

1. The tested gangue still contained little coal. Here we ignited the prepared samples at temperature of 650 °C for 8 h in muffle oven.
2. We took 0.5 g of each ignited sample into a Teflon cup, and added small amount of ultra-pure water into the sample to make it wet. Then we added concentrated hydrofluoric acid, concentrated nitric acid and per-chloric acid and placed it on a hot plate for digestion.
3. Until the sample had dissolved completely, we transferred the solution to a 25 mL volumetric flask for constant volume.
4. Then the digestion solutions were sent for analyses of trace metal concentrations by using Inductively Coupled Plasma Mass Spectrometry (ICP-MS), produced by Thermo-Fisher ©, its detection limits and relative standard deviation (RSD) of trace elements were shown as follows in Table 1. This device has high analysis precisions for testing of heavy mass elements.

### 2.4. Dynamic Leaching Study of Backfill Materials

Backfill materials are mixtures of coal gangue and fly ash, the fly ash is dominated by fine particles, with similar properties of adsorptive clay, high content of trace metal elements and poor water solubility; for gangue material, it has high proportion of clay minerals, relative low content of trace metal elements and small proportion of soluble components. Natural leaching is the main process for the migration of trace metal elements into environment. During the leaching process, elements’ releasing depends not only on their absolute content, but also on their occurrence states. Through the method of soil column dynamic leaching experiment, the present situation and transformation of trace metal elements’ occurrence states under natural precipitation actuation could be studied. Soil column dynamic leaching method could be used for risk assessment of this method in different circumstances. It is an important method to study migration and state transformation of pollutants in soil and groundwater. It is widely used in research fields such as water conservancy, agriculture and soil science [40,41,42].

**Figure 2:**
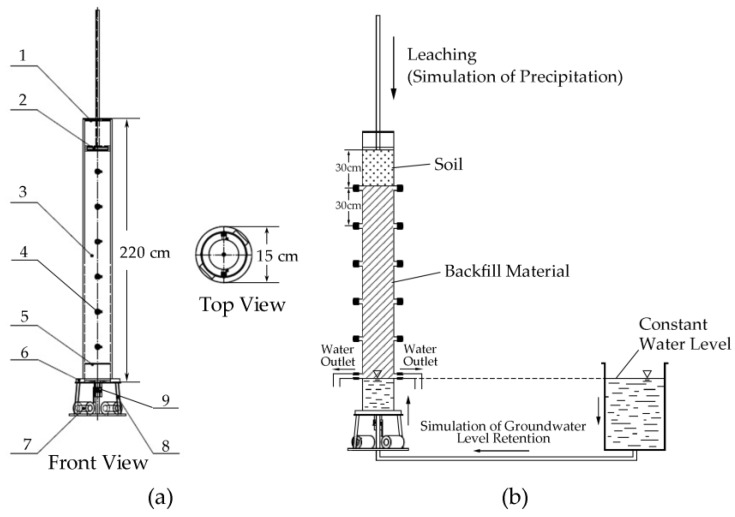
Testing Soil Column: (**a**) structure of soil column: 1-Testing soil column; 2-Piston rod assembly with graduated line; 3-Acrylic tube; 4-Angle valve; 5-Permeable tray; 6-Acrylic tube; 7-Balancing weight; 8-Base assembly; 9-Ball valve; (**b**) installation of soil column.

The soil column used in this experiment was made of polymethyl methacrylate (PMMA) material, which has high stability and corrosion resistance. The height of the column was 220 cm, the cross-sectional diameter was 15 cm, and the cross-sectional area was 176.71 cm^2^. The bottom of the soil column is connected with iron weights for stabilization. There is a permeable plate above the column bottom with a distance of 10 cm. In addition to the screws between the base and the column body, it is also equipped with a seal to make it watertight. At the top of the column, there is a piston rod with scale lines, which has a water inlet. On both sides of the column body, 6 water outlet angle valves are uniformly installed from top to bottom, the distance between 2 angle valves is 30 cm. On the bottom of the column there is a ball valve for water inlet or leachate outlet. The structure of the soil column is shown in Figure 2a as follows.

Water seepage from top to bottom and water saturating from bottom to top are common methods of dynamic leaching. According to the condition of studying area, there is groundwater which saturated the backfill material at the bottom and there is also natural precipitation which seepages water from top to bottom. Hence we fill the column with backfill material to be a height of 180 cm, and then on the top of backfill we filled natural soil with a height of 30 cm. For dynamical leaching of backfill material we made the bottom two angle valves open and through the bottom ball valve to insure 30 cm backfill material always be submerged in water at column bottom, which simulated the effect of groundwater. From the top we simulated the natural precipitation to enter column through the piston rod according to local daily rainfall of 2017. The total experiments were implemented for 1 year, with simulated the real atmospheric precipitation of 2017. The testing period was from the beginning of January 2017 to the end of December 2017. Four soil columns was filled with designed backfill materials as coal gangue and fly ash proportions of 1:0.2, 1:0.4, 1:0.6, 1:0.8 were tested. The installation of the soil column dynamical leaching experiment is shown in Figure 2b as follows. The tested samples of backfill material after leaching experiment were sampled about 10 mm above the simulated groundwater level, at the bottom of the soil column.

**Figure 3:**
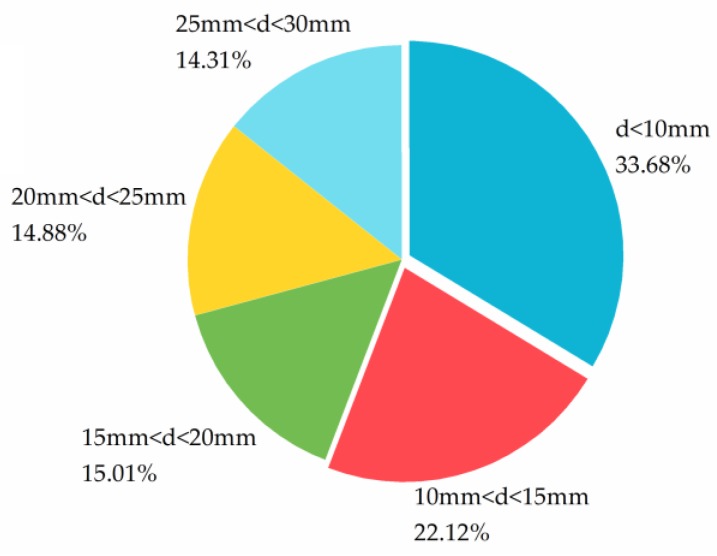
Coal gangue particle size distribution (mass).

Coal gangues were sampled from the top, middle and bottom of the dump from Quantai–Qishan coalmine, sampled gangues were sieved according to the fractions of 10 mm, 15 mm, 20 mm, 25 mm and 30 mm, respectively. The particle size distribution is shown in Figure 3 as follows.

The tested amount of backfill materials which were filled in each soil column are shown in Table 2 as follows:

At the inlet and outlet of valves, steel wire screens and glass fiber with a thickness of 1 cm were used to prevent the runoff loss from fine material particles. Peristaltic pumps were used for water inlet according to the daily precipitation.

### 2.5. Sequential Extraction Analysis

The laboratory equipment used in this study include: Insmark-IP126 type pH meter, Shanghai Lu Xiangyi TG1650-WS high-speed centrifuge, Changzhou Nuoji HY-5 rotary oscillator, Shanghai Haoyu FB224 automatic electronic analytical balance, Shanghai temperature control instrument factory DHG-9023A constant temperature drying oven, Crystal SY-1230 constant temperature water bath, 50 mL centrifuge tube, etc. Experimental reagents include: deionized water, ammonium acetate, hydroxylamine chloride, 30% hydrogen peroxide solution, nitric acid, sodium acetate, perchloric acid, etc.

The sequential extraction experiments were implemented as follows (modified according to our study purpose and previous studies [43]):

Crushing the backfill material samples before and after column leaching experiment until it could pass through the 200-mesh sieve. Then took 100 g of each crushed samples for analysis. For each analysis, 0.5 g sample was needed. The extraction processes are:

(1) *Exchangeable state fraction*: The room temperature was about 25 °C. We took 0.5 g (accuracy about 0.1 mg) of sample and place it in a 50 mL centrifuge tube, 10 mL of 1 mol/L ammonium acetate were added and then adjust the pH value to 7 with nitric acid. Then we put it in a mechanical shaker for 2 h (HY-5 orbital shaker). The resulting solution was centrifuged at 3500 rpm for 15 min (TG1650-WS high-speed centrifuge). The supernatant was then removed with a pipette and stored for analysis. Then the remaining residue in the centrifuge tube was washed with 20 mL deionized water and centrifuged at 3500 rpm for 15 min. (2) *Carbonate binding state fraction*: We put the remaining solids from the step (1) in a centrifuge tube, added 10 mL of 1 mol/L sodium acetate, and then adjusted the pH-value to 5 with nitric acid. Then, it was mixed in a mechanical shaker for 6 h, and we put the solution to stand over one night. Then the solution was centrifuged at 3500 rpm for 15 min. The following steps were the same as in step (1). (3) *Iron-manganese combined state fraction*: 20 mL of 0.1 mol/L ammonium hydrogen chloride were added to the remaining solids from step (2) in centrifuge tube, and then adjusted the pH-value to 2 with nitric acid. Then we put it in a water bath which was set at 85 °C for 6 h (SY-1230 constant temperature water bath kettle). During the water bath heating, we shook it occasionally. Then we let the solution to stand overnight and then were centrifuged at 3500 rpm for 15 min. The supernatant was removed and collected for further steps. The remaining solids were washed again as described in step (1). (4) *Organic sulfide binding state fraction*: To the remaining solids from step (3), 5 mL of 30% (mass) H_2_O_2_ were added and the mixture was stirred manually at room temperature for 1 h, and then put it in a water bath which was set at 85 °C for 1 h to reduce the volume fraction; then added 5 mL of 30% (mass) H_2_O_2_ solution again, and repeated the water bath step to evaporate and digest until they were nearly dry; after cooling, 25 mL of 1 mol/L ammonium acetate were added; then adjusted the pH-value to 2 with nitric acid, and mixed them in a mechanical shaker for 8 h. The solution was then centrifuged at 3500 rpm for 15 min and put it to stand overnight. The supernatant was then removed with a pipette into 50 mL volumetric flask and stored for analysis. The remaining steps were the same as step (1). (5) *Residual state fraction*: To the remaining solids from step (4), 6 mL 6 mol/L HF, 2 mL 6 mol/L HNO_3_ and 2 mL 6 mol/L HClO_4_ were added and allow the mixture stand overnight; then put it in a water bath for set at 90 °C for 2 h. The resulting solution was then centrifuged at 3500 rpm for 15 min, then the supernatant was removed with a pipette into 50 mL volumetric flask and stored for analysis.

In this research, if the tested backfill materials have high enough homogeneities, according to the digestion analysis, total content of a specific trace element includes above mentioned 4 fractions and the residual fraction. Then we could calculate with subtraction method, i.e., residual state = total amount − (exchangeable state + carbonate binding state + iron-manganese combined state + organic sulfide binding state).

## 3. Results

### 3.1. Results of Sequential Extraction before Leaching

As we have mentioned in Section 2.3, the studied trace metal concentrations in gangue, fly ash and four proportioned backfill materials are shown in Table 3 as follows.

The Cu concentrations ranged from 76.63 to 231.62 mg/kg, with the lowest value occurring in the full gangue and the highest value in the full fly ash. The Zn concentrations ranged from 243.02 to 422.57 mg/kg, with the lowest value occurring in the 1:0.2 proportion and the highest value in the full fly ash. The Pb concentrations were in the range of 57.57 to 103.37 mg/kg, the lowest value was found in the full gangue, and the highest value was in the full fly ash. The Cr concentrations ranged from 153.78 to 556.82 mg/kg, with the lowest value occurred in the full gangue and the highest value in the full fly ash. The concentrations of Cd were in the range of 0.63 to 1.60 mg/kg, the lowest value was found in the full gangue, and the highest value was found in full fly ash. The Mn concentrations ranged from 614.72 to 1433.10 mg/kg, with the lowest value occurred in the full fly ash and the highest value in the full gangue. The Ni content ranged from 81.93 to 96.54 mg/kg, with the lowest value occurred in the full fly ash and the highest value in the full gangue.

**Figure 4:**
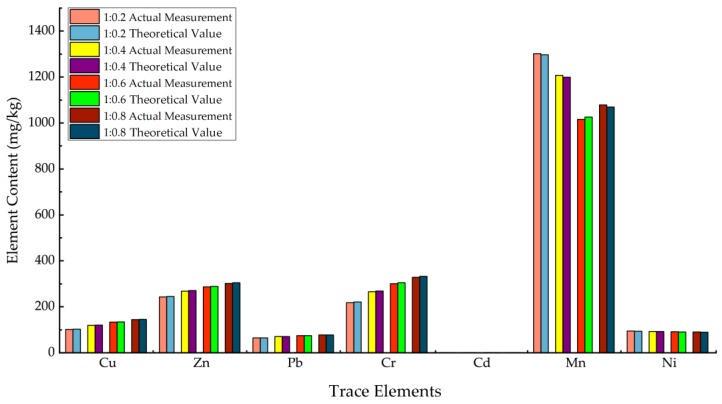
Comparison of the actual trace metal and theoretical concentrations in backfill material through digestion analysis (before leaching). (Cd: Actual measurement: 0.78; 0.9; 0.98; 1.05. Theoretical value: 0.79; 0.91; 0.99; 1.06.).

The tested results of full coal gangue and full fly ash are also shown in Table 3. To verify the homogeneity of backfill materials with 4 different proportions, we calculated their theoretical trace elements’ content by using tested corresponding contents in full gangues and full fly ashes, as well as the proportions between used gangue and fly ash i.e., the 1:0.2, 1:0.4, 1:0.6 and 1:0.8. Then we measured the actual trace elements’ content in different backfill materials also by using digestion analysis. Finally, we compared both results which are shown in Figure 4 as follows:

Compared with the theoretical concentrations, the deviation between the measured values and theoretical value of tested trace metal contents in all 4 kinds of backfill materials were only about 1%, which means that the raw material samples had high homogeneity and the results of the above-mentioned digestion analysis were reliable. The results could be used for further analysis together with sequential extraction.

The results of sequential extraction analysis are shown as follows from Table 4, Table 5, Table 6 and Table 7.

It could be deduced that for the tested trace elements, the residue states and the organic sulfide binding states were accounted as the main parts among all occurrence states, over 65% in mass. In which the sum of these two states of 1:0.2 proportion was 68%, in 1:0.4 proportion was 61%, in 1:0.6 proportion was 60%, and in 1:0.8 proportion was 67%. In addition, the sum of these two states of full gangue was 65%, in full fly ash was 72%. The two active states, i.e., the exchangeable state and the carbonate binding state of tested trace metals had very low proportions in mass, accounted for 4.97% and 11.01%, respectively. Due to the low concentration of these two active states, the trace metal elements in the backfill materials had only limited risks to the environment. The occurrence states of Cr show that, Cr was mainly in the residue state with a mean value nearly 93%. The lowest value of 91% occurred in the 1:0.2 proportion, and the highest value of 94% appeared in the 1:0.8 proportion. The proportion of its residual state increases with the proportion of fly ash. The occurrence states of Cd show that the iron-manganese combined state and the organic sulfide binding state were accounted for more than 50% of all kinds of materials, the average proportion of the two states was up to 55%. Both the exchangeable state and the residue state increased with an increasing of fly ash proportion. The iron-manganese combined state fell sharply between 1:0.6 and 1:0.8 proportions. The carbonate binding state and the organic sulfide binding state decreased as the proportion of fly ash increased, but they also increased sharply between proportions 1:0.6 and 1:0.8. Probably there is a certain inflection point between the proportions of 1:0.6 and 1:0.8. The occurrence states of Cu shows that the sum of the carbonate binding state, the organic sulfide binding state and the residual state in the respective proportions occupied about 94% in mass, mainly in the organic sulfide binding state, followed by carbonate binding state and residue state. Due to strong ability of Cu in forming complex, the resulted chelates had strong stability. The occurrence states of Mn element shows that the iron-manganese combined state was dominant among all proportions, with a mean value approximately 62%, then followed by residue state and organic sulfide binding state. Exchangeable and carbonate binding states were very few, with a total proportion less than 10%. The occurrence state of Ni element shows that among all 4 kinds of materials, the organic sulfide binding state and residual state were dominate, with a sum about 68% in mass. The proportions of exchangeable, carbonate binding, and iron-manganese combined states were low, only 8%, 13%, and 12% in mass, respectively. The occurrence state of Pb shows that among all 4 kinds of materials, residue states were dominant, with an average proportion of 70% in mass, followed by 19% of organic sulfide binding state. Exchangeable and carbonate binding states accounted for only about 1%. The occurrence states of Zn element shows that in each kind of material, organic sulfide binding states were dominant, totally accounted for about 39%. Followed by iron-manganese combined state and residue state, each accounted for 26%. The residue state increases with the content of fly ash.

The experimental results show that seven trace metals which were studied appeared all five occurrence states. For Cr and Pb, residual state occupied the main part; for Mn, Cd and Zn, iron-manganese combined state took the main part; and for Cu, carbonate binding state accounted for the main part; for Ni, organic sulfide binding state and residual state occupied the main part. The harmful trace metal elements, which are mainly composed of residual state and organic sulfide binding state, have limited risk to environment.

### 3.2. Results of Sequential Extraction after Leaching

Digestion analyses after soil column leaching experiments were implemented on the backfill materials including proportions of 1:0.2, 1:0.4, 1:0.6, and 1:0.8. The results are shown in Table 8.

Cu concentrations ranged from 172.49 to 298.85 mg/kg, with the lowest value occurred in 1:0.6 proportion and the highest value in 1:0.4 proportion. Zn concentrations ranged from 520.11 to 796.74 mg/kg, with the lowest value occurring in 1:0.6 proportion and the highest value in 1:0.4 proportion. Pb concentrations were in the range of 98.97 to 117.38 mg/kg, the lowest value was found in 1:0.6 proportion, and the highest value was in 1:0.4 proportion. Cr concentrations ranged from 243.16 to 271.05 mg/kg, with the lowest value occurred in 1:0.2 proportion and the highest value in 1:0.8 proportion. The concentrations of Cd were in the range of 1.21 to 1.70 mg/kg, the lowest value was found in 1:0.6 proportion, and the highest value was found in 1:0.8 proportion. Mn concentrations ranged from 592.08 to 922.77 mg/kg, with the lowest value occurred in 1:0.4 proportion and the highest value in 1:0.6 proportion. Ni contents ranged from 85.56 to 95.31 mg/kg, with the lowest value occurred in 1:0.6 proportion and the highest value in 1:0.2 proportion.

Results of sequential extraction experiments after leaching are shown in Table 9, Table 10, Table 11 and Table 12 as follows.

Among all 4 kinds of tested materials, after leaching the residual state and the Organic sulfide binding state still accounted for the major parts of the total occurrence state, and the sum of these two states exceeded 78% in mass. In addition, in 1:0.2 proportion the sum of these two states was 82% in mass, the sum of these two states in 1:0.4 proportion was 79%, in 1:0.6 proportion was 77%, and in 1:0.8 proportion was 78%. Compared with the materials before leaching, the sum of the residual state and the organic sulfide binding state among the tested trace metals increased with an average of 23%. The exchangeable state and carbonate binding state were kept in low level, accounted for 3.89% and 9.30%, respectively, and there was a slight decrease compared with the backfill material before leaching. After leaching the migration ability of harmful trace metals in the backfill material had been further restricted to a certain extent. After leaching the total amount of Cu and Pb were significantly higher than that before leaching. Especially Cu increased by maximum of 169% in 1:0.2 proportion. The total amount of Ni element also increased a little after leaching, varied between 1% and 6%. After leaching the total amount of Zn was also higher than before, with an average increase rate up to 140%. After leaching the total amount of Cr in 1:0.2 proportion increased by 11% compared to before leaching. After leaching the total amount of Cd was significantly higher than that before leaching, with average increasing of 61%. After leaching the total amount of Mn was significantly reduced compared to that before leaching. The average decline rate achieved 39%.

The increasing of total amount of tested trace metals shows that under the driving of hydrodynamic effect by leaching. The overall migration of trace metal was to be transported deeper inside the backfill material, i.e., to go deeper inside the reclaimed subsidence area. Meanwhile the occurrence states of the tested metal elements had become more and more stable to against hydro-migration effect.

### 3.3. Sequential Extraction Analysis Before and After Soil Column Leaching

**Figure 5:**
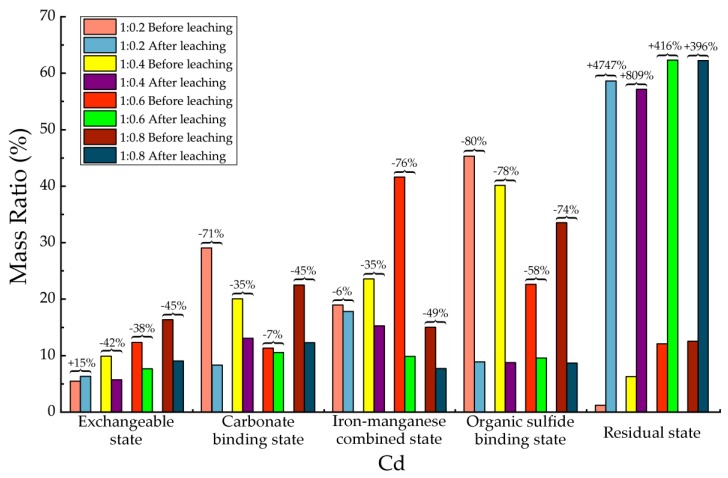
Sequential extraction of Cd before and after leaching in different occurrence states.

**Figure 6:**
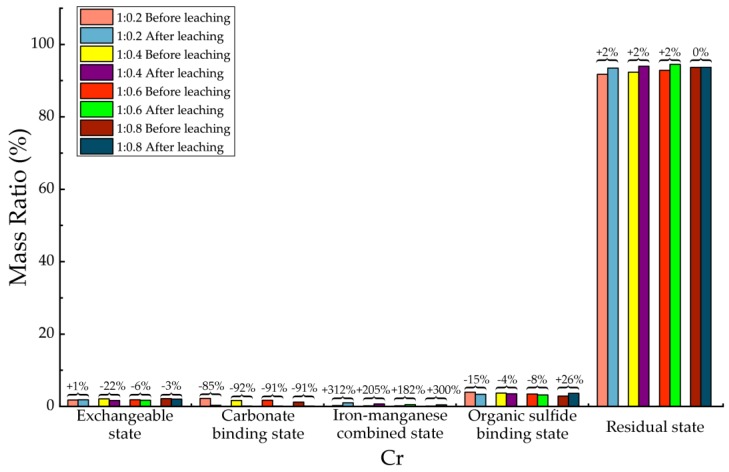
Sequential extraction of Cr before and after leaching in different occurrence states.

**Figure 7:**
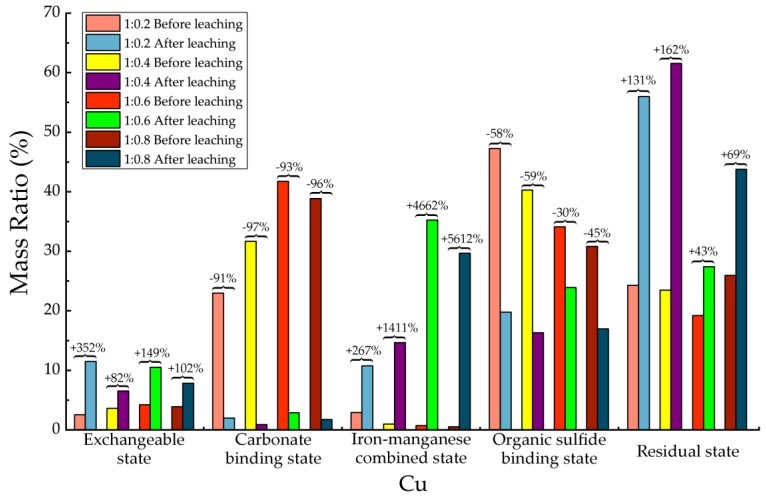
Sequential extraction of Cu before and after leaching in different occurrence states.

**Figure 8:**
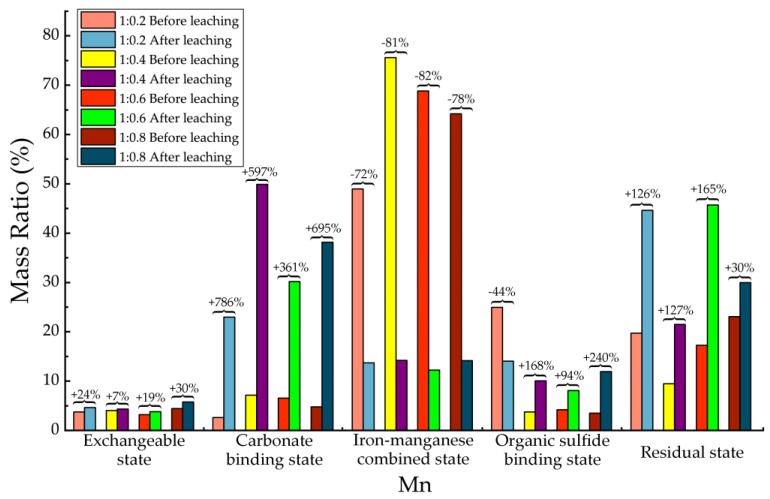
Sequential extraction of Mn before and after leaching in different occurrence states.

**Figure 9:**
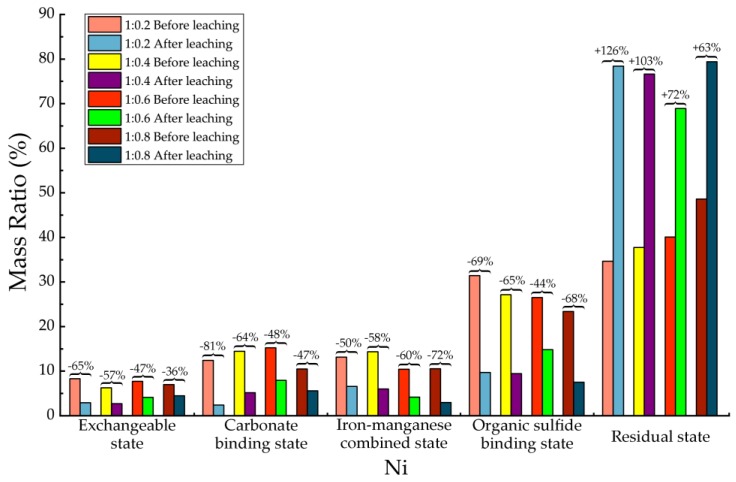
Sequential extraction of Ni before and after leaching in different occurrence states.

**Figure 10:**
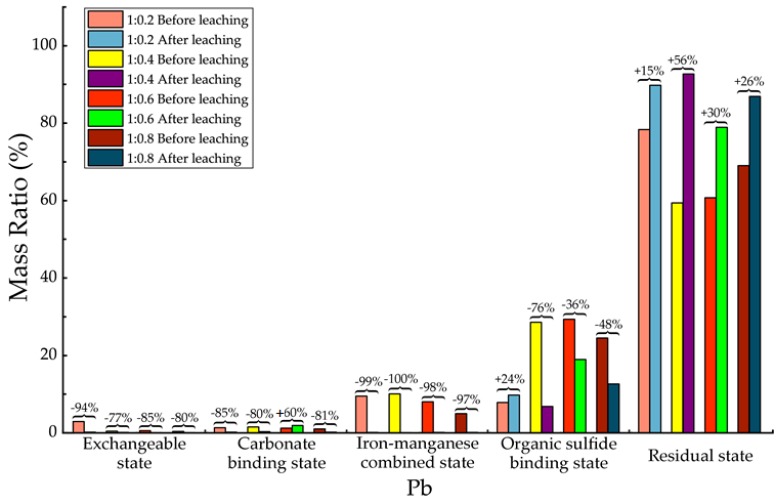
Sequential extraction of Pb before and after leaching in different occurrence states.

**Figure 11:**
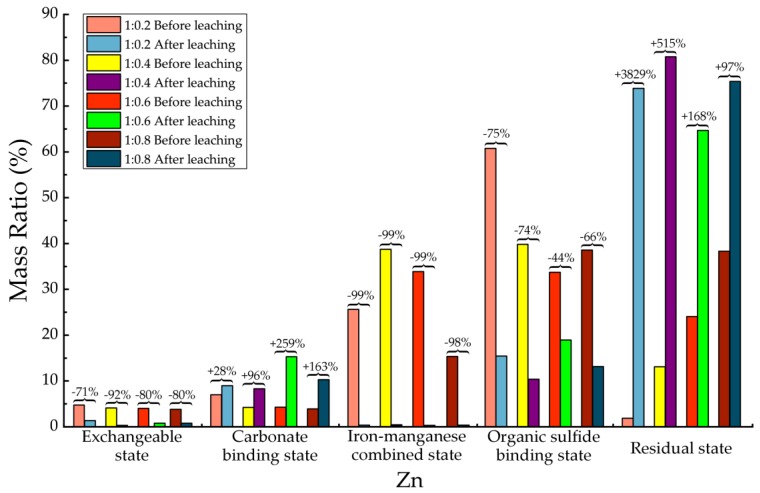
Sequential extraction of Zn before and after leaching in different occurrence states.

Before and after soil column leaching experiments, the trace metals’ occurrence states of Cd, Cr, Cu, Mn, Ni, Pb and Zn were analyzed, which are shown in Table 13, Table 14, Table 15, Table 16, Table 17, Table 18 and Table 19, as well as Figure 5, Figure 6, Figure 7, Figure 8, Figure 9, Figure 10 and Figure 11 as follows, respectively.

According to Figure 5 and Table 13, after leaching the most significant change of Cd was the increasing of residue state concentration, which up to 1600% on average among all 4 proportions. In addition, meanwhile, other occurrence states’ decreased significantly. The concentration of exchangeable state decreased about 38–42% among all proportions except its concentration of 1:0.2 proportion, which was a little bit higher than that before leaching, which could due to experimental errors. The concentration decreasing of carbonate binding Cd was 7–71% among all proportions. The highest decreasing was found in 1:0.2 proportion. The concentration decreasing of iron-manganese combined Cd was 6–76%. In addition, the concentration decreasing of organic sulfide-binding Cd ranged from 58% to 80%. From the results, it could be deduced that after leaching, the occurrence states of Cd had become much more inactive. Its migration abilities in all states were completely decreased and the residual state were significantly increased to a large extent. Furthermore, considering the absolute amount of Cd in backfill material was very low, we could make the conclusion that the Cd has nearly no potential of environmental risks.

According to Figure 6 and Table 14, there was no significant change in the exchangeable state, organic sulfide binding state and residual state of Cr before and after leaching. Their variations in concentration ranged from 0% to 26%. The most significant change happened in iron-manganese combined state of Cr, with average increasing among all 4 proportions about 250% after leaching. Then followed by the carbonate binding state, whose concentration decreased by more than 85% after leaching. The results had demonstrated that in backfill materials, most of the Cr element existed as residual state. The Exchangeable state, carbonate binding state, iron-manganese combined state and organic sulfide binding state occupied only very few proportion, and the acting of hydrodynamic leaching has very limited effect on transformation of Cr’s occurrence state, only significant effects on transformation of carbonate binding state to iron-manganese combined state, which had also decreased the migration ability of Cr to a certain extent.

According to Figure 7 and Table 15, after leaching the concentrations of the exchangeable state, the iron-manganese combined state, and the residual state of Cu were significantly increased. Especially the average increasing of iron-manganese combined states was up to 3000%, in which the maximum increasing was up to 5612% in 1:0.8 proportion. The exchangeable state increased with an average of 171% after leaching, with a maximum value of 352% in 1:0.2 proportion. After leaching the average increasing of residual state was 101%, with the highest value in 1:0.4 proportion of 162%. The concentration of carbonate binding state and organic sulfide binding state in all 4 proportions were significantly reduced after leaching. The carbonate binding state decreased with an average of 94%. In addition, the concentration of organic sulfide binding state decreased averagely about 48%. For Cu element in backfill material, most of them were transformed from a carbonate binding state to iron-manganese combined state, as well as from organic sulfide binding state to residual state. Although the exchangeable state of Cu was also increased to a certain extent, consider the absolute quantity among all occurrence states, the overall migration activity of Cu element had been significantly restricted.

According to Figure 8 and Table 16, after leaching the proportions of carbonate binding state, residue state, and exchangeable state of the Mn increased. The carbonate binding state was obviously increased than before leaching, with an average of 607%. The increase of residual state was relatively higher than that before leaching, with an average of 112%t. For organic sulfide binding state, its content in 1:0.2 proportion decreased than before leaching, but in the other proportions it increased. For iron-manganese combined state, its content in each proportion decreased significantly than before leaching, with an average decrease of 78%, from which the maximum decrease was 82% in 1:0.6 material, then 72% in 1:0.2 material.

According to Figure 9 and Table 17, for Ni element, after leaching only the concentration of residue state increased significantly. The concentrations of other states decreased. Among all 4 kinds of materials, the average increasing of residual state was 91% in mass than before leaching. The exchangeable state decreased about 36% to 65% after leaching, and the carbonate binding state decreased 47–81%. The decreasing of iron-manganese combined state ranged from 50% to 72% and the organic sulfide binding state decreased 44–69%. After soil column leaching, the exchangeable state, carbonate binding state, iron-manganese combined state as well as the organic sulfide binding state of Ni in backfill material were significantly decreased and the residual state increased greatly, which demonstrated that the potential of Ni transportation into ambient environment had been strictly decreased.

According to Figure 10 and Table 18, for Pb element, after leaching there was almost only the residual state increased significantly. The concentration of other 4 states decreased, among which the exchangeable state decreased about 77–94% in mass. The decreasing of carbonate binding state ranged from 80% to 85%, but for the proportion of 1:0.6 there was an increasing of 60% which is unreasonable. Consider the absolute amount of Pb carbonate binding state, the reason might due to experimental error. The decreasing of iron-manganese combined state ranged around 99%. The decreasing of organic sulfides states ranged from 36% to 78%, and there was also a small increasing of 24% in the proportion of 1:0.2, which had no influence the overall trend of decreasing. The experimental results demonstrated that after leaching Pb element exited in backfill material mainly in residual state and a little bit organic sulfide binding state, the activity of Pb element in migration from backfill material to ambient environment had been greatly restricted.

According to Figure 11 and Table 19, for Zn element, after leaching the carbonate binding state and residual state increased significantly. The residual state increased with an average of 1152% in mass. The increasing of carbonate binding state was 137% on average. The proportions of the other three states were all significantly decreased, especially the iron-manganese combined state was almost eliminated, with an average decreasing of 99%. The decreasing of exchangeable state was 71–92%. In addition, the decreasing of the organic sulfide binding state ranged from 44% to 75%. The results demonstrated that after leaching most Zn element exited in backfill material mainly as residual state, a little bit organic sulfide binding state, and few carbonate binding states. Most of the Zn element had been significantly restricted in migration under hydrodynamic leaching.

## 4. Discussion

Dai et al. have pointed out that, according to the quality of coal resources in Xuzhou, environmental risks exist during the whole life cycle of coal production and use because of the contents of trace metals [2]. In addition, in Xuzhou, former reclaimed mining subsidence areas by using only gangue material, the content of trace metals like Cd, Cr, Cu, Pb, etc. exceeded China soil quality standards [4,5,7]. In this work, we presented a novel method and case study to restrict the migration ability of trace metal elements in gangues by using another kind of coalmine solid waste—fly ashes from coal combustion. This study verified that after leaching the residual states of Cd, Cr, Ni and Zn in backfill materials was greatly increased than before leaching; the residual state and carbonate binding state of Mn were also significantly increased while its iron-manganese combined state decreased greatly. The residual state of Pb also increased to a certain extent after leaching, and its iron-manganese combined state decreased to almost none; the carbonate binding state of Cu after leaching was significantly decreased, while its iron-manganese combined state and residual state increased greatly. After leaching, Cd, Ni, Zn, Cr, and Pb are significantly restricted to be released into the environment than that before leaching, and Cu, Mn could still have little opportunities to migrate in reclamed land base.

Under dynamical leaching from natural precipitation, Cd, Cr, Ni, Pb and Zn in backfill material were mainly presented in residual state, which demonstrated that these trace metal elements were significantly restricted in migration and they have low risks to environment. This finding has good agreement with previous studies. Matong et al. reported that in the soil of Free State, South Africa, Cd was found to perform more actively than other trace elements, with main reason of anthropogenic industries instead of minerals’ releasing [44], and like the results in this study, trace elements with inert mineral binding state had low migration ability. Yang et al. reported in Xinhua coalmine area of Guizhou, China, the main occurrence state of tested trace elements was also residual state, except Cd, which also performs more active than other trace elements because the local gangues were easily to be weathered, and weathering effect could increase the activity of Cd. However, in our backfill material design, air could be effectively isolated by using fine fly ashes, to avoid weathering effect [45]. Bian et al. pointed out that with the weathering and leaching effect in mining rocks, the pH could be decreased and the trace elements Zn, Cu, Cd, Cr, and Pb were released in to environment. Lime material was suitable to reduce the acidity [1,27]. In our study, by using fly ash, the same effect as with lime could be achieved. Zhou et al. reported that in Huainan city which locates about 300 km south of Xuzhou, the situation was most of the trace elements were strongly associated with sulfide minerals, which could also be easily released under oxidation and leaching effect, only leaching effect also had low potential of environmental risks [46]. According to our research and previous research, the avoidance of comprehensive effect by oxidation and natural leaching is important, gangues with adequate fly ash backfilled land area could prevent air from permeation, and the reconstructed overlying soil layer could also separate the reclaimed land base with air, therefore our method has low risks to the environment.

According to study results, Mn and Cu still have potential risk to the environment, since the carbonate binding state, iron-manganese combined state and organic sulfide binding state were still major remnants. As Sungur et al. has mentioned that in Turkey, once these elements had entered overlying soil, these states can be oxidized easily under the natural conditions and migrate into the environment [47]. Another previous study from Tang et al. reported that in the Huainan coalmine area, Mn could be a moderate threat to the environment, since the iron-manganese combined state were more easily to be oxidized than other states [48], here we have had very similar results, Mn still had migration potential due to increasing of carbonate binding state. In addition, Matong et al. have also reported similar results [44]. Previous studies have also indicated that trace metals could cause pollutions to soil under strong acidic conditions, especially with participation of sulfide minerals. However, in our research, alkaline fly ash was used to modify gangue, which could keep the reclaimed land base in alkaline states and restrict the migration risks. O’Connor et al. also reported that with the decreasing of pH, the adsorption of trace metals in soil also decreased and further led to migration [15]. Zhou et al. also revealed that Cd has low risk to environment in alkaline condition [46], which supports our results. Sungur et al. revealed that Pb possessed high mobility and consequent more availability in acid soluble and reducible fractions, which means that Pb can cause environmental pollution easily [47], but in our research, residue state of Pb took up almost 90%, other fractions except organic sulfides state were basically none, since fly ash brought the avoidance of acidity in reclaimed land. Wang et al. reported a novel efficient method by using MgO, cement and ground granulated blast-furnace slag as binding agent for trace metals solidification in soil, especially for Cu, Zn, and Ni, which also supported the relationship between pH values and leachability of trace metals, this results also supported our method by using fly ash, which is similar to cement with certain contents of MgO [20,21].

Generally, isolation of coalmine wastes from air i.e., weathering and oxidation effect of natural environment is also critical to restriction of trace element migration. Gangues modification with fly ash could fulfill this condition. In this research we studied seven environmentally sensitive trace metals, some other elements like mercury (Hg), arsenic (As), which could also cause serious environment pollutions were not studied, since they are rare found in local strata of Xuzhou Qishan-Quantai coalmine area. Trace elements like Mn and Cu were still not restricted significantly, in future works, more trace elements, more proportioning of backfill materials from different coalmines should be tested. Yin et al. reported by using flay ash in reclamation, it could have influence on physical structure of reconstructed overlaying soil, by using sludge and fungi could restore the soil aggregations and the soil organic carbon [6]. Therefore in future studies we should also considered the optimization of soil quality, at the same time of contamination control.

## 5. Conclusions

Through this study, it has been verified that the hydrodynamic migration activity of tested seven trace metals Cu, Zn, Pb, Cd, Cr, Mn and Ni in gangue-fly ash backfill materials had been significantly restricted. Under the driving of natural leaching effect of precipitation, although the migration of the tested trace metal elements was downstream, which went always deeper inside the backfill reclaimed land base of subsided area, the occurrence state of most trace metal elements was transformed to be much more inactive in releasing and migration. Among all tested trace metal elements, Mn performed different from others, which was mainly transformed from iron-manganese combined state to carbonate binding state, it could lead to a little bit increasing in its migration ability. Hence in a further study, specific agents to inhibit activity of Mn element could be added into the backfill material. Gangue-fly ash backfill materials with proportions of 1:0.2 and 1:0.4 performed high transformation efficiency of trace metals from active states to inert states than proportion of 1:0.6 and 1:0.8, which meant that excessive fly ash has negative effects on occurrence state transformation. Cd was found to have nearly no potential risks, and Cu was found to be bond to carbonates, Fe and Mn oxides as well as organic sulfides, both results are in good agreement with relative studies, such as Marquez et al.

The simulated groundwater level in this research was −2 m, which was much higher than in the local area. With the increasing of groundwater depth, with the increasing of migration distance and period, more trace metals are believed to be transformed in inert and in active states. However, there was still a certain amount of trace metals existed in iron-manganese combined state and organic sulfide binding state, these two states might be activated if the pH-value of groundwater changed. In old coalmine waste dumps, acid leachate is always produced with the inside infiltration of oxygen. Hence the reconstructed overlying soil layer must have appropriate thickness, in order to prevent oxygen from infiltration and avoid the producing of acid leachate. Gangue and fly ash are easily acquired in coalmine area, by using gangue-fly ash backfill materials it can solve both gangue dumping and land subsidence problems. In future, based on this case study, gangue-fly ash backfill materials from more coalmine areas in China should also be studied.

## Figures and Tables

**Table 1:** Detection limits and RSD of the applied ICP-MS.

Elements	Light Mass Elements	Medium Mass Elements	Heavy Mass Elements
Detection limits (μmol/kg)	0.013–2.927	0.053–0.413	0.043–0.090
RSD (%)	0.6–3.3	0.8–1.2	1.0–2.1

**Table 2:** Coal gangue and fly ash filled in soil column. Unit: Kg.

Mass Ratio/Material	Gangue	Fly Ash
1:0.2	25.00	5.00
1:0.4	22.43	8.97
1:0.6	20.75	12.45
1:0.8	19.22	15.37

**Table 3:** Digestion test results for all kinds of materials (before leaching). Unit: mg/kg.

Proportion/Element	Cu	Zn	Pb	Cr	Cd	Mn	Ni
1:0.2	101.57 ± 2.18	243.02 ± 4.33	64.96 ± 0.79	218.62 ± 1.79	0.78 ± 0.01	1302.04 ± 17.07	94.23 ± 1.80
1:0.4	119.59 ± 2.29	267.91 ± 3.02	70.28 ± 0.69	265.47 ± 2.36	0.9 ± 0.07	1206.93 ± 24.68	92.53 ± 2.03
1:0.6	133.24 ± 1.95	286.78 ± 1.95	74.32 ± 1.53	300.96 ± 2.87	0.98 ± 0.04	1015.53 ± 21.50	91.26 ± 1.62
1:0.8	143.93 ± 3.37	301.54 ± 3.19	77.48 ± 0.79	328.75 ± 3.11	1.05 ± 0.04	1078.74 ± 25.98	90.26 ± 0.94
Full gangue	76.63 ± 1.12	286.66 ± 1.64	57.57 ± 0.97	153.78 ± 2.10	0.63 ± 0.03	1433.10 ± 26.83	96.54 ± 1.33
Full fly ash	231.62 ± 2.13	422.57 ± 3.54	103.37 ± 1.78	556.82 ± 3.92	1.60 ± 0.06	614.72 ± 12.29	81.93 ± 1.86

**Table 4:** Results of sequential extraction of 1:0.2 samples. Unit: mg/kg.

Occurrence State	Trace Elements Contents						
	Cu	Zn	Pb	Cr	Cd	Mn	Ni
Exchangeable state	2.58	11.49	1.90	3.95	0.04	48.75	7.83
Carbonate binding state	23.34	17.02	0.88	4.87	0.23	33.78	11.73
Iron—manganese combined state	2.98	62.34	6.17	0.55	0.15	637.61	12.40
Organic sulfide binding state	48.02	147.60	5.11	8.59	0.36	325.01	29.62
Residual state	24.65	4.56	50.90	200.66	0.01	256.88	32.65

**Table 5:** Results of sequential extraction of 1:0.4 samples. Unit: mg/kg.

Occurrence State	Trace Elements Contents						
	Cu	Zn	Pb	Cr	Cd	Mn	Ni
Exchangeable state	4.30	10.99	0.30	5.64	0.09	48.75	5.79
Carbonate binding state	37.89	11.32	1.07	4.35	0.18	86.46	13.40
Iron—manganese combined state	1.16	103.72	7.06	0.58	0.21	912.29	13.30
Organic sulfide binding state	48.19	106.73	20.08	9.73	0.36	45.40	25.13
Residual state	28.06	35.15	41.76	245.17	0.06	114.03	34.92

**Table 6:** Results of sequential extraction of 1:0.6 samples. Unit: mg/kg.

Occurrence State	Trace Elements Contents						
	Cu	Zn	Pb	Cr	Cd	Mn	Ni
Exchangeable state	5.62	11.48	0.44	5.49	0.12	32.54	7.05
Carbonate binding state	55.62	12.24	0.90	5.21	0.11	66.41	13.89
Iron—manganese combined state	0.99	97.20	6.00	0.50	0.41	699.11	9.53
Organic sulfide binding state	45.43	96.78	21.84	10.42	0.22	42.36	24.19
Residual state	25.58	69.08	45.15	279.34	0.12	175.11	36.60

**Table 7:** Results of sequential extraction of 1:0.8 samples. Unit: mg/kg.

Occurrence State	Trace Elements Contents						
	Cu	Zn	Pb	Cr	Cd	Mn	Ni
Exchangeable state	5.58	11.52	0.31	7.02	0.17	47.96	6.33
Carbonate binding state	55.92	11.78	0.81	3.97	0.24	51.77	9.45
Iron—manganese combined state	0.74	46.22	3.87	0.37	0.16	692.29	9.53
Organic sulfide binding state	44.33	116.41	19.00	9.52	0.35	37.90	21.10
Residual state	37.35	115.62	53.49	307.86	0.13	248.82	43.85

**Table 8:** Digestion results of 4 kinds of materials (after leaching). Unit: mg/kg.

Proportion/Element	Cu	Zn	Pb	Cr	Cd	Mn	Ni
1:0.2	273.51 ± 4.20	665.45 ± 6.84	112.76 ± 4.86	243.16 ± 4.10	1.49 ± 0.02	656.58 ± 6.62	95.31 ± 1.76
1:0.4	298.85 ± 3.82	796.74 ± 8.20	117.38 ± 2.04	268.47 ± 3.80	1.49 ± 0.04	592.08 ± 7.56	91.19 ± 0.59
1:0.6	172.49 ± 3.97	520.11 ± 9.87	98.79 ± 1.06	258.85 ± 0.64	1.21 ± 0.02	922.77 ± 13.12	85.56 ± 1.27
1:0.8	259.16 ± 1.64	675.23 ± 6.15	112.26 ± 2.45	271.05 ± 2.58	1.70 ± 0.05	613.2 ± 6.23	92.25 ± 2.04

**Table 9:** Results of sequential extraction of 1:0.2 samples (after leaching). Unit: mg/kg.

Occurrence State	Trace Elements Contents						
	Cu	Zn	Pb	Cr	Cd	Mn	Ni
Exchangeable state	31.40	8.98	0.19	4.43	0.09	30.53	2.79
Carbonate binding state	5.38	59.74	0.23	0.83	0.12	150.75	2.29
Iron—manganese combined state	29.43	2.50	0.12	2.50	0.27	90.00	6.26
Organic sulfide binding state	54.14	102.65	10.98	8.16	0.13	92.28	9.24
Residual state	153.16	491.57	101.24	227.25	0.88	293.03	74.73

**Table 10:** Results of sequential extraction of 1:0.4 samples (after leaching). Unit: mg/kg.

Occurrence State	Trace Elements Contents						
	Cu	Zn	Pb	Cr	Cd	Mn	Ni
Exchangeable state	19.55	2.50	0.12	4.48	0.09	25.60	2.47
Carbonate binding state	2.71	66.06	0.35	0.36	0.20	295.56	4.71
Iron—manganese combined state	43.81	2.09	0.04	1.81	0.23	84.05	5.48
Organic sulfide binding state	48.78	82.81	8.04	9.44	0.13	59.64	8.63
Residual state	184.00	643.28	108.83	252.38	0.85	127.24	69.90

**Table 11:** Results of sequential extraction of 1:0.6 samples (after leaching). Unit: mg/kg.

Occurrence State	Trace Elements Contents						
	Cu	Zn	Pb	Cr	Cd	Mn	Ni
Exchangeable state	18.13	4.16	0.09	4.43	0.09	35.04	3.53
Carbonate binding state	5.00	79.64	1.92	0.38	0.13	278.52	6.80
Iron—manganese combined state	60.79	1.55	0.14	1.24	0.12	112.65	3.57
Organic sulfide binding state	41.28	98.46	18.69	8.22	0.12	74.77	12.70
Residual state	47.29	336.31	77.97	244.58	0.76	421.80	58.96

**Table 12:** Results of sequential extraction of 1:0.8 samples (after leaching). Unit: mg/kg.

Occurrence State	Trace Elements Contents						
	Cu	Zn	Pb	Cr	Cd	Mn	Ni
Exchangeable state	20.26	5.30	0.09	5.61	0.15	35.35	4.13
Carbonate binding state	4.49	69.57	0.22	0.29	0.21	234.02	5.15
Iron—manganese combined state	76.97	2.31	0.16	1.19	0.13	86.91	2.77
Organic sulfide binding state	44.01	88.90	14.21	9.91	0.15	73.10	6.95
Residual state	113.43	509.15	97.59	254.06	1.06	183.82	73.25

**Table 13:** Mass ratio variation of Cd occurrence states before and after leaching.

State of Occurrence/Proportion	1:0.2	1:0.4	1:0.6	1:0.8
Exchangeable state	15%	−42%	−38%	−45%
Carbonate binding state	−71%	−35%	−7%	−45%
Iron—manganese combined state	−6%	−35%	−76%	−49%
Organic sulfide binding state	−80%	−78%	−58%	−74%
Residual state	4747%	809%	416%	396%

**Table 14:** Mass ratio variation of Cr occurrence states before and after leaching.

State of Occurrence/Proportion	1:0.2	1:0.4	1:0.6	1:0.8
Exchangeable state	1%	−22%	−6%	−3%
Carbonate binding state	−85%	−92%	−91%	−91%
Iron—manganese combined state	312%	205%	182%	300%
Organic sulfide binding state	−15%	−4%	−8%	26%
Residual state	2%	2%	2%	0%

**Table 15:** Mass ratio variation of Cu occurrence states before and after leaching.

State of Occurrence/Proportion	1:0.2	1:0.4	1:0.6	1:0.8
Exchangeable state	352%	82%	149%	102%
Carbonate binding state	−91%	−97%	−93%	−96%
Iron—manganese combined state	267%	1411%	4662%	5612%
Organic sulfide binding state	−58%	−59%	−30%	−45%
Residual state	131%	162%	43%	69%

**Table 16:** Mass ratio variation of Mn occurrence states before and after leaching.

State of Occurrence/Proportion	1:0.2	1:0.4	1:0.6	1:0.8
Exchangeable state	24%	7%	19%	30%
Carbonate binding state	786%	597%	361%	695%
Iron—manganese combined state	−72%	−81%	−82%	−78%
Organic sulfide binding state	−44%	168%	94%	240%
Residual state	126%	127%	165%	30%

**Table 17:** Mass ratio variation of Ni occurrence states before and after leaching.

State of Occurrence/Proportion	1:0.2	1:0.4	1:0.6	1:0.8
Exchangeable state	−65%	−57%	−47%	−36%
Carbonate binding state	−81%	−64%	−48%	−47%
Iron—manganese combined state	−50%	−58%	−60%	−72%
Organic sulfide binding state	−69%	−65%	−44%	−68%
Residual state	126%	103%	72%	63%

**Table 18:** Mass ratio variation of Pb occurrence states before and after leaching.

State of Occurrence/Proportion	1:0.2	1:0.4	1:0.6	1:0.8
Exchangeable state	−94%	−77%	−85%	−80%
Carbonate binding state	−85%	−80%	60%	−81%
Iron—manganese combined state	−99%	−100%	−98%	−97%
Organic sulfide binding state	24%	−76%	−36%	−48%
Residual state	15%	56%	30%	26%

**Table 19:** Mass ratio variation of Zn occurrence states before and after leaching.

State of Occurrence/Proportion	1:0.2	1:0.4	1:0.6	1:0.8
Exchangeable state	−71%	−92%	−80%	−80%
Carbonate binding state	28%	96%	259%	163%
Iron—manganese combined state	−99%	−99%	−99%	−98%
Organic sulfide binding state	−75%	−74%	−44%	−66%
Residual state	3829%	515%	168%	97%
